# Triphenylamine‐Based Porous Organic Polymers with High Porosity: their High Carbon‐Dioxide Adsorption and Proton‐Conductivity Emergence

**DOI:** 10.1002/smll.202410794

**Published:** 2025-02-17

**Authors:** Kohei Okubo, Showa Kitajima, Hitoshi Kasai, Kouki Oka

**Affiliations:** ^1^ Institute of Multidisciplinary Research for Advanced Materials Tohoku University 2‐1‐1 Katahira, Aoba‐ku Sendai Miyagi 980‐8577 Japan; ^2^ Carbon Recycling Energy Research Center Ibaraki University 4‐12‐1 Nakanarusawa Hitachi Ibaraki 316‐8511 Japan; ^3^ Deuterium Science Research Unit Center for the Promotion of Interdisciplinary Education and Research Kyoto University Yoshida Sakyo‐ku Kyoto 606‐8501 Japan

**Keywords:** carbon dioxide adsorption, Iodine, porosity, porous organic polymers, proton conductivity

## Abstract

Amorphous porous organic polymers (**POP**s) feature high specific surface area and chemical and thermal stability; therefore, they are applied in various fields. It is previously reported that chemical polymerization using iodine as an oxidant enables the synthesis of amorphous **POP**s without impurities. In this study, an iodine‐based chemical polymerization method is employed to maximize the specific surface area of polytriphenylamine, a typical amorphous **POP**. Furthermore, 1,3,5‐tris[4‐(diphenylamino)phenyl]benzene, a monomer with three triphenylamine moieties connected by a benzene core, is used to increase the number of reaction points and construct a rigid structure. The resulting poly[1,3,5‐tris[4‐(diphenylamino)phenyl]benzene] (**pTTPA**) exhibited a high specific surface area. Using 200 equivalents of iodine resulted in a **pTTPA** with the largest Brunauer–Emmett–Teller (**BET**) specific surface area (2134.6 m^2^ g^−1^) among previously reported triphenylamine‐based amorphous **POP**s, and demonstrated a high CO_2_ adsorption capacity (3.31 mmol g^−1^ at 25 °C). Furthermore, **pTTPA** exhibited significant water–vapor adsorption when the **BET** specific surface area reached 1500 m^2^ g^−1^, leading to the emergence of proton conductivity (e.g., 4.33 × 10^−6^ S cm^−1^ at 95% RH and 90 °C). The findings demonstrate that iodine‐based chemical polymerization enables the maximization of the porosity of amorphous **POP**s and the development of proton conductivity within them.

## Graphical abstract

The porosity of polytriphenylamine, a typical amorphous porous organic polymer (**POP**s), is maximized via an iodine‐based chemical polymerization. The resulting amorphous **POP**s exhibited significant water–vapor adsorption with increasing specific surface area, leading to the emergence of proton conductivity. Furthermore, high CO_2_ adsorption capacity is also observed. This study promotes the development of novel functionalities of amorphous **POP**s.

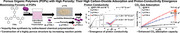

## 1 Introduction

Porous organic polymers^[^ ^1^ ^]^ (**POP**s) are porous materials composed entirely of organic molecules and have attracted considerable attention owing to their composition of earth‐abundant elements and the facile modulation of their pore shapes and functions^[^ ^2^ ^]^ based on their molecular design. Furthermore, as **POP**s do not contain metals, **POP**s are lightweight and feature low density, allowing them to achieve higher specific surface area than those of other inorganic or organic–inorganic hybrid porous materials.^[^ ^3^ ^]^ Among **POP**s, amorphous **POP**s,^[^ ^4^ ^]^ constructed through irreversible covalent bonds, exhibit superior chemical and thermal stability than those of crystalline **POP**s, also known as covalent organic frameworks,^[^ ^5^ ^]^ which are formed through reversible covalent bonds.

The functionality of porous materials, including **POP**s, emerges at the interface between the framework and the adsorbate; therefore, the functionality is considerably affected by their specific surface area.^[^ ^6^ ^]^ An increase in surface area promotes the adsorption and diffusion of substances,^[^ ^7^ ^]^ contributing to an enhanced adsorption capacity for gas molecules,^[^ ^3^, ^8^ ^]^ water vapor,^[^ ^9^ ^]^ and volatile organic compound vapors.^[^ ^3b^ ^]^ Strategies to enhance the specific surface area of amorphous **POP**s include constructing highly porous structures via copolymerization using monomers of different shapes or sizes,^[^ ^10^ ^]^ as well as constructing rigid structures, which are likely to maintain their porosity, by increasing the degree of polymerization.^[^ ^11^ ^]^ Furthermore, although many amorphous **POP**s are synthesized through chemical polymerization,^[^ ^12^ ^]^ such as oxidative polymerization and cross‐coupling reactions, residual metals^[^ ^13^ ^]^ from the oxidants and catalysts have been reported to remain in the resultingg amorphous **POP**s, clogging the pores^[^ ^14^ ^]^ and leading to reduced specific surface area.^[^ ^15^ ^]^ Therefore, the synthesis of a highly porous structure without impurities is crucial for constructing amorphous **POP**s with a high specific surface area.

This study focuses on polytriphenylamine,^[^ ^12^, ^16^ ^]^ a representative amorphous **POP**, and attempts to maximize its porosity. Especially, we employed 1,3,5‐tris[4‐(diphenylamino)phenyl]benzene (**TTPA**) as a monomer because the size of the monomer is expanded by the connection of three triphenylamine units with the central benzene ring and it has many reaction points, facilitating the formation of a highly porous and rigid structure. Moreover, we employed an iodine‐based chemical polymerization method^[^ ^17^ ^]^ that uses iodine as an easily removable oxidant^[^ ^18^ ^]^ instead of a metal salt. This method enables the synthesis of impurity‐free amorphous **POP**s and facilitates the construction of a highly porous structure.

## 2 Results and Discussion

### 2.1 Synthesis and Characterization of pTTPA

**Figure 1:**
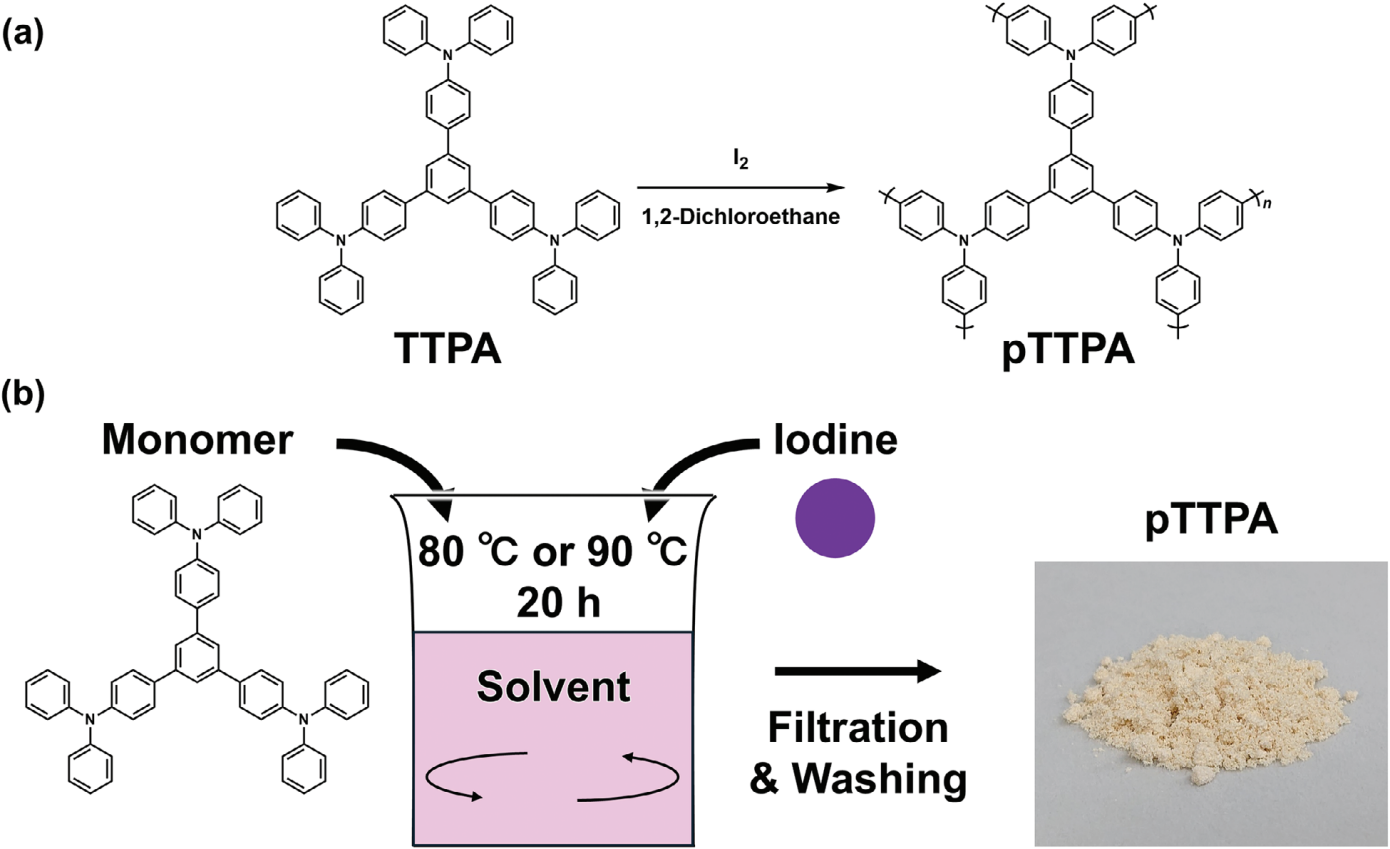
Schematics of a) **pTTPA** polymerization and b) procedures for solution polymerization.

As shown in **Figure** **1**, **TTPA** was polymerized by stirring it and iodine in 1,2‐dichloroethane. Specifically, as shown in **Table** **1**, to investigate the influence of reaction conditions on the resulting polymer, the iodine amount and reaction temperature varied. After polymerization, the precipitated **pTTPA** was collected via filtration and washed with ethanol to obtain pale‐yellow **pTTPA** powders, denoted as **pTTPA**s **1–7**.

**Table 1:** Synthesis of **pTTPA** under different reaction conditions.

Sample^a)^	Amount of iodine [mmol]	Temperature [°C]
pTTPA 1	10	80
pTTPA 2	40	80
pTTPA 3	100	80
pTTPA 4	200	80
pTTPA 5	500	80
pTTPA 6	200	90
pTTPA 7	500	90

^a)^ Reaction condition: **TTPA** (1 mmol) and iodine in 1,2‐dichloroethane (33 mL) for 20 h.

**Figure 2:**
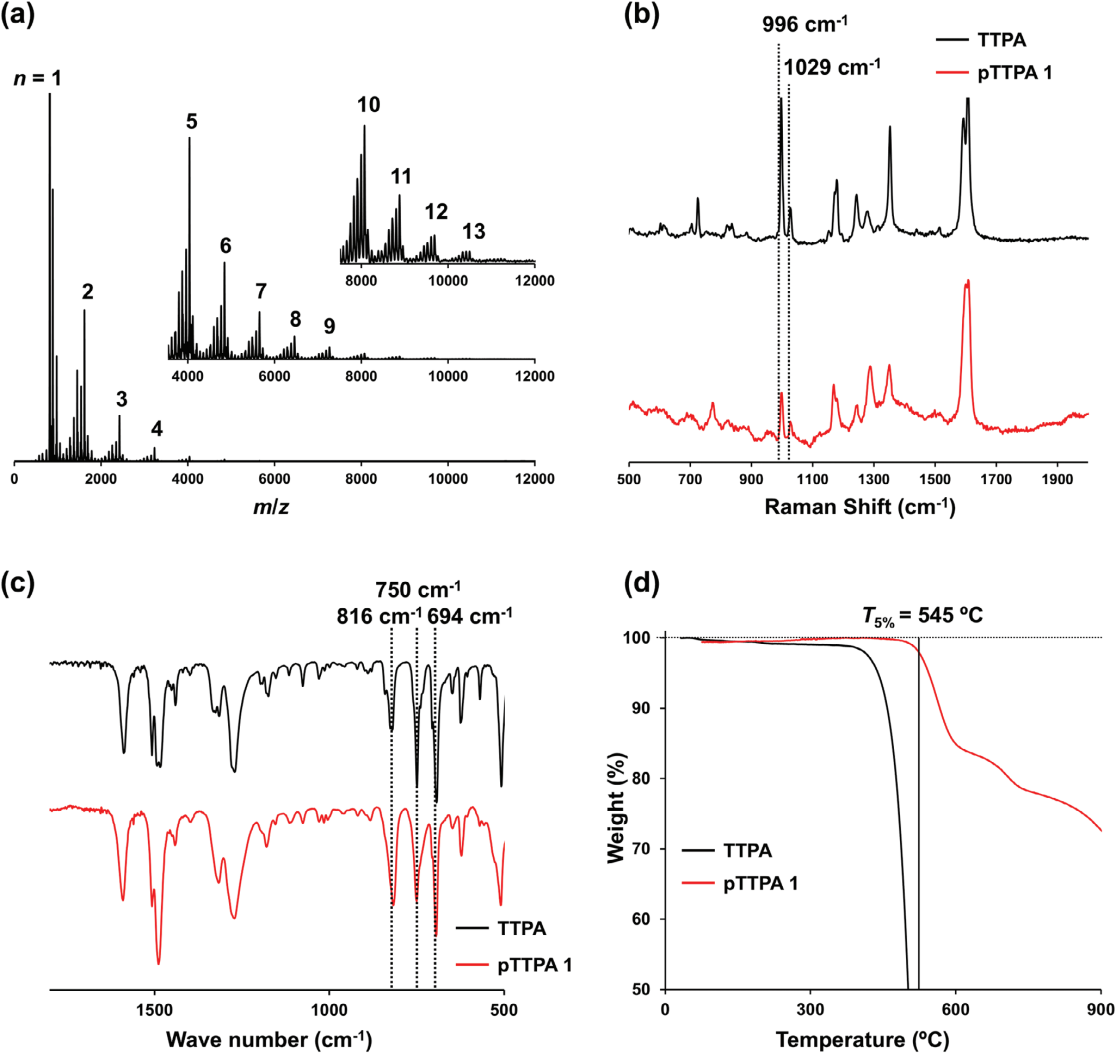
a) **MALDI‐TOF MS** spectra of **pTTPA 1**. *n* refers to the number of **TTPA** units in the polymer; therefore, the difference between the main segment peaks is 808.0. The peaks among the main segment peaks indicate fragmentation between C─N bonds.^[^ ^23^ ^]^ For **pTTPA**s **2–7**, which were expected to be more polymerized than pTTPA1, a higher laser power than that of **pTTPA 1** was required for ionization. This likely caused carbonization during laser ablation,^[^ ^24^ ^]^ preventing the acquisition of **MALDI‐TOF MS** spectra for **pTTPA 2–7**. b) Raman spectra, normalized by the peaks at 1610 cm^−1^ (C═C stretching vibration of benzene), c) **IR** spectra, normalized by the peaks at 1590 cm^−1^ (C═C stretching vibration of benzene), and d) **TG** curves of **TTPA** (black) and **pTTPA 1** (red).

The progress of polymerization was confirmed by matrix‐assisted laser desorption ionization‐time of flight (**MALDI‐TOF**) mass spectroscopy (**MS**), solid‐state ^13^C nuclear magnetic resonance (**NMR**), Raman spectroscopy, infrared (**IR**) spectroscopy, and thermogravimetric (**TG**) analysis. **Figure** **2**a–c shows the results for **pTTPA 1** as a representative example. As shown in Figure 2a, the **MALDI‐TOF MS** spectrum indicates the formation of polymers comprising ≥13 **TTPA** units.

As shown in Figures S1 and S2 (Supporting Information), the solid‐state ^13^C NMR spectra exhibit peaks at *δ* = 126, 137, and 146 ppm, corresponding to unsubstituted aromatic carbons, substituted aromatic carbons, and the carbon bonded to the N atom of the triphenylamine moiety,^[^ ^19^ ^]^ respectively. These peaks are present in both the **TTPA** and **pTTPA 1** spectra, indicating that the core chemical structure remains largely unchanged after polymerization. Furthermore, the peak corresponding to the para position of the benzene ring on the triphenylamine unit of **TTPA** (Figure S1, Supporting Information) is reduced in **pTTPA 1**, supporting the progress of polymerization.

As shown in Figure 2b, the Raman spectrum reveals a reduction in the peaks associated with the C–H bending vibration (996 cm^−1^) and ring deformation vibration (1029 cm^−1^) of **TTPA** in **pTTPA 1**.^[^ ^20^ ^]^ This observation supports the consumption of the monosubstituted benzene on the triphenylamine unit during the reaction. Then, as shown in Figure 2c, the **IR** spectrum shows a decrease in the peak corresponding to the C–H bending vibration (694 cm^−1^, 750 cm^−1^) of the monosubstituted benzene of the triphenylamine moiety in **TTPA** and the emergence of a new peak at 816 cm⁻¹ associated with the C–H bending vibration of 1,4‐disubstituted benzene.^[^ ^20^ ^]^ These changes indicate a decrease in monosubstituted benzene and the generation of 1,4‐disubstituted benzene on the triphenylamine. These changes in the two spectra Figure 2b,c) further confirm that polymerization progressed at the para position of the benzene ring on the triphenylamine unit.

As shown in Figure S3 (Supporting Information), the rate of decrease in absorbance at the C–H bending vibration (694 cm^−1^) of monosubstituted benzene in the **IR** spectrum was used to semi‐quantify the polymerization degree for each condition. In Table S2 (Supporting Information), the comparisons of **pTTPA**s **1–5** supported that the reaction progressed faster with increasing amounts of iodine. However, the reaction rate (Table S2, Supporting Information, right) slowed down significantly when the amount of iodine exceeded 200 equivalents per molecule of **TTPA**. Furthermore, a comparison of **pTTPA**s **4** and **6** supported that an increase in reaction temperature slightly enhanced the reaction rate. Conversely, a comparison of **pTTPA**s **5** and **7** indicates that when 500 equivalents of iodine are used per molecule of **TTPA**, increasing the temperature had no effect on the reaction rate. These comparisons suggest that **pTTPA**s **5–7** exhibited similar reaction rates and polymerization degrees, which were higher than those of the other samples.

Additionally, the thermal stability of the polymers was evaluated via **TG** analysis under a N_2_ atmosphere, and Figure 2d shows the results for **pTTPA 1** as a representative example. The 5% weight loss temperature (*T*_5%_) of **pTTPA 1** was 545 °C, supporting the formation of the polymer and exhibiting high thermal stability comparable to or exceeding that of conventional amorphous **POP**s containing triphenylamine.^[^ ^21^ ^]^ As shown in Figure S4 (Supporting Information), **pTTPA 6**, which is expected to be more polymerized than **pTTPA 1**, exhibited a similar *T*_5%_ (543 °C) to **pTTPA 1**, whereas the weight loss ratio below 600 °C (12%) was lower than that of **pTTPA 1** (16%). These results indicate that the weight loss below 600 °C is derived from the decomposition of the easily decomposed terminal triphenylamine moiety,^[^ ^22^ ^]^ supporting that **pTTPA 6** has a lower ratio of terminal triphenylamine moieties than **pTTPA 1** owing to the higher polymerization degree.

Furthermore, in Figure S5 (Supporting Information), the scanning electron microscopy with energy dispersive X‐ray spectroscopy (**SEM‐EDX**) spectrum of **pTTPA 1** shows that there are no peaks corresponding to the L‐line of iodine or other impurities, such as metals, indicating that residual impurities in **pTTPA 1** were below the detection limit of 0.1 atm.%; this was also confirmed for the other samples. As shown in Figure S6 (Supporting Information), the powder X‐ray diffraction (**PXRD**) pattern of **pTTPA 1** exhibits no distinct peaks, confirming the formation of an amorphous structure through irreversible covalent bonding.

**Figure 3:**
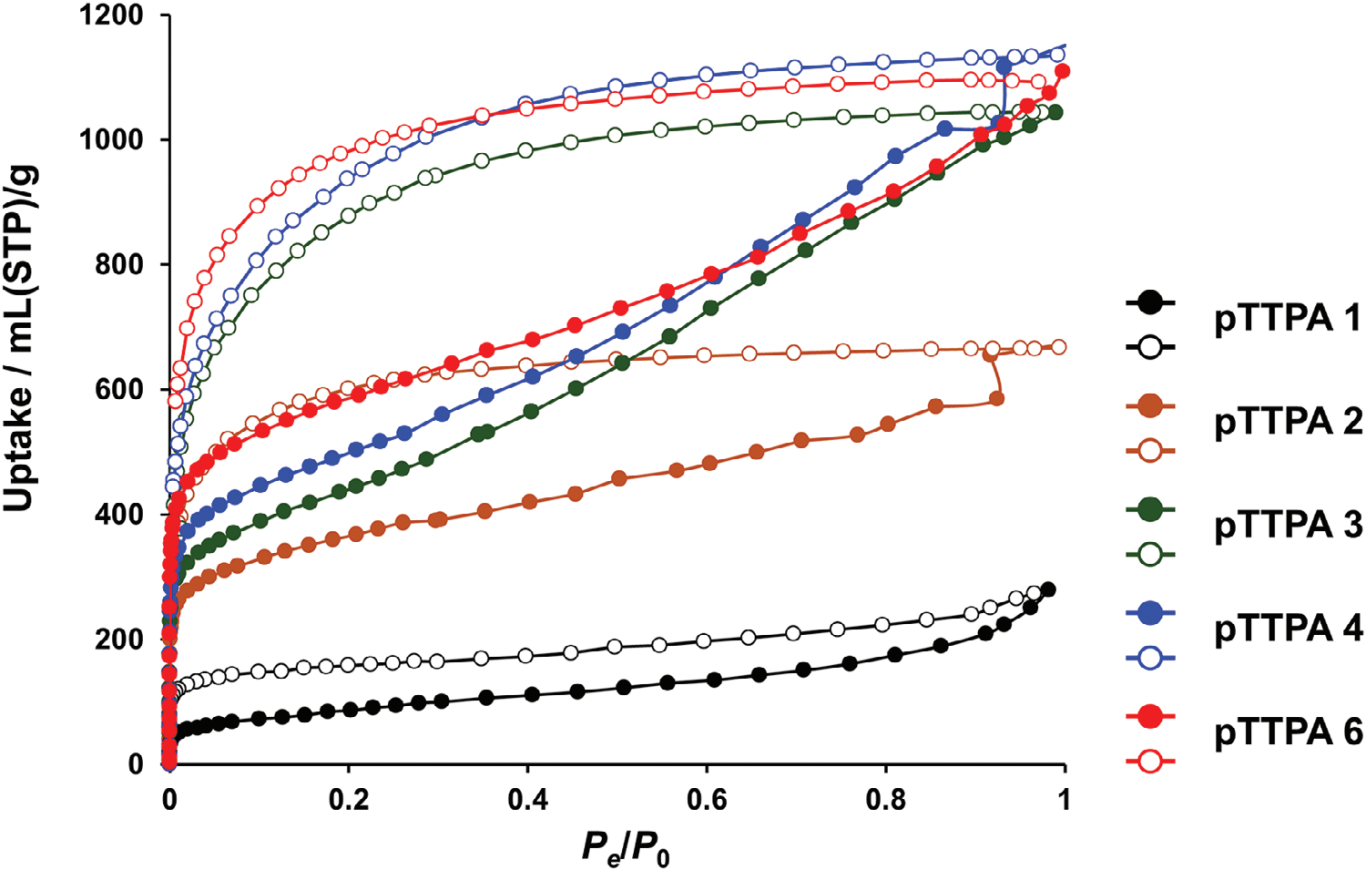
N_2_ adsorption isotherms of **pTTPA 1** (black), **pTTPA 2** (brown), **pTTPA 3** (green), **pTTPA 4** (blue), and **pTTPA 6** (red), measured at 77 K. Solid and hollow symbols denote the adsorption and desorption isotherms, respectively.

As shown in **Figures** **3** and S7 (Supporting Information), N_2_ adsorption measurements of **pTTPA**s **1–7** were conducted at 77 K to calculate the Brunauer–Emmett–Teller (**BET**) specific surface area (***S*_BET_**); the results are summarized in **Table** **2**. As shown in Table 2, ***S*_BET_** increased as the reaction progressed, reaching a maximum of 2134.6 m^2^ g^−1^ for **pTTPA 6**. Specifically, ***S*_BET_** increases as the polymerization progresses because the pore size decreases, allowing for easy maintenance of the highly porous structure.^[^ ^11^ ^]^ Notably, no significant changes were observed in the ***S*_BET_** of **pTTPA**s **5** and **7**, which were likely to have a similar degree of polymerization. As shown in Table S3 (Supporting Information), **pTTPA 6** exhibited the highest ***S*_BET_** among triphenylamine‐based amorphous **POP**s. Notably, as shown in Figure 3, all **pTTPA**s exhibited a swelling phenomenon,^[^ ^25^ ^]^ characterized by significant hysteresis in adsorption and desorption isotherms. Furthermore, as shown in Figure S8 (Supporting Information), **pTTPA**s **2** and **4** exhibited a gate‐opening phenomenon,^[^ ^26^ ^]^ wherein the adsorption amount increased sharply at a specific relative pressure. These phenomena arise from the structural changes that occur during the adsorption of gas molecules and support the high flexibility of **pTTPA**. In **pTTPA**s **3** and **5–7**, which had similar degrees of polymerization to **pTTPA 4**, the gate‐opening phenomenon was not observed. This is presumably because **pTTPA**s **3** and **5–7** were still undergoing swelling even at a relative pressure of 1.0, which was the upper limit of the measurement range, and these gate pressures were above the measurement range. The adsorption amounts of **pTTPA**s **3** and **5–7** continued to increase even at relative pressures ≥ 0.9, without reaching a plateau^[^ ^27^ ^]^ indicative of adsorption saturation or capillary condensation^[^ ^28^ ^]^ indicative of pore filling, supporting the aforementioned assumption.

**Table 2:** Polymerization degrees and pore‐structure parameters of **pTTPA**s **1–7**. The polymerization degree was indicated as the rate of decrease from the monomer **TTPA** in the absorbance corresponding to the C–H bending vibration of monosubstituted benzene in the **IR** spectrum (Figure S3, Supporting Information), which decreases according to the progress of polymerization.

Sample	Polymerization degree [%]	*S*_BET_ ^a)^ [m^2^g^−1^]	*S*_tot_ ^b)^ [m^2^g^−1^]	*S*_micro_ ^c)^ [m^2^g^−1^]	*V*_micro_ ^d)^ [cm^3^g^−1^]
pTTPA 1	17	315.93	301.63	195.5	0.082
pTTPA 2	53	1318.2	1451.5	1250.0	0.681
pTTPA 3	57	1579.8	1691.8	1476.0	1.23
pTTPA 4	59	1802.8	2026.8	1973.3	1.51
pTTPA 5	60	1976.6	2195.5	1898.7	1.17
pTTPA 6	60	2134.6	2297.9	2029.8	1.17
pTTPA 7	60	1984.1	2238.1	1956.6	1.11

^a)^ ***S*_BET_**: **BET** specific surface area;

^b)^ ***S*_tot_**: total surface area calculated by t‐plots method;

^c)^ ***S*_micro_**: micropore surface area calculated by t‐plots method;

^d)^ ***V*_micro_**: micropore volume calculated by t‐plots method.

### 2.2 Proton Conductivity of pTTPAs

**Figure 4:**
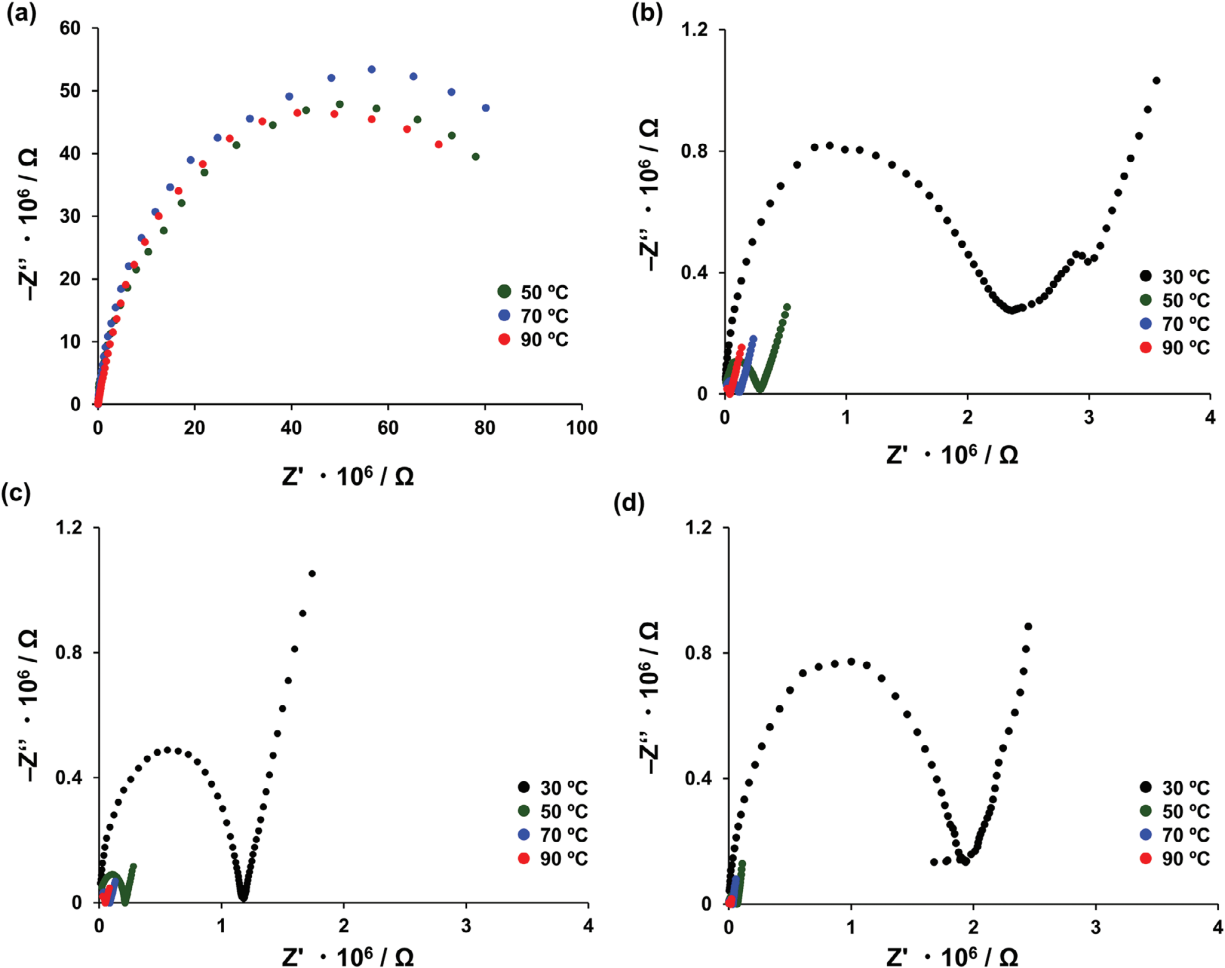
Proton conductivities **pTTPA** a) **1**, b) **3**, c) **4**, and d) **6**. Impedance spectra of the disk‐shaped pellets of each **pTTPA** under 95% RH at 30 °C (black), 50 °C (green), 70 °C (blue), and 90 °C (red).

As shown in **Figure** **4**, the proton conductivities of the pelletized **pTTPA**s **1**, **3**, **4**, and **6** were measured under 95% RH at various temperatures via electrochemical impedance spectroscopy. As shown in Figure 4a, **pTTPA 1** exhibited a negligible proton conductivity of approximately 10^−10^ S cm^−1^ under all conditions. By contrast, as shown in Figure 4b–d, the proton conductivities of **pTTPA 3**, **4,** and **6** increased drastically with increasing temperature and at 90 °C under 95% RH, they reached 1.34 × 10^−6^, 4.33 × 10^−6^, and 3.64 × 10^−6^ S cm^−1^, respectively, representing an increase of four orders than that of **pTTPA 1** under the same conditions. Figure S9 (Supporting Information) shows an Arrhenius plot of **pTTPA 4** under 95% RH at various temperatures, indicating an activation energy of 0.52 eV for proton conduction, suggesting that proton conductivity is induced through a preferential contribution of the vehicle mechanism.^[^ ^29^ ^]^ Water molecules confined in a nanoscale space exhibit a decreased activation barrier for self‐dissociation, facilitating the formation of hydronium ions, which act as proton carriers.^[^ ^30^ ^]^ Because **pTTPA** has no hydrophilic functional groups in its framework, the interaction between the generated hydronium ions and the framework is expected to be weak.^[^ ^9^ ^]^ Consequently, the mobility of hydronium ions in the pores is increased,^[^ ^29b^ ^]^ promoting proton conduction via the vehicle mechanism. The observed proton conductivities and activation energy were comparable to those of other **POP**s^[^ ^31^ ^]^ which have no proton‐donating functional groups, such as sulfonic groups.

**Figure 5:**
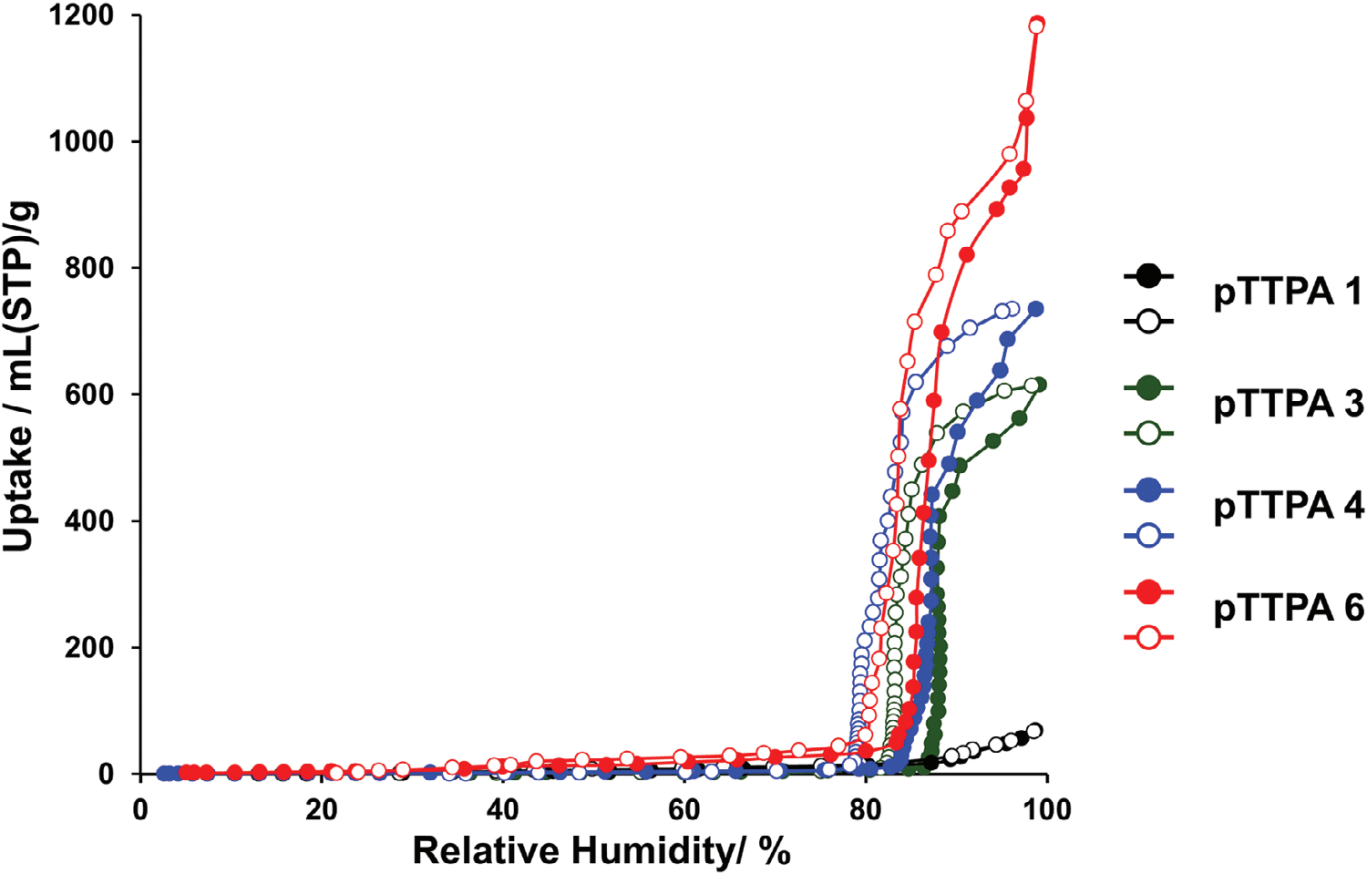
Water–vapor adsorption isotherms of **pTTPA 1** (black), **pTTPA 3** (green), **pTTPA 4** (blue), and **pTTPA 6** (red), measured at 298 K. Solid and hollow symbols denote the adsorption and desorption isotherms, respectively.

As shown in **Figure** **5**, to clarify the significant differences in the proton conductivities of **pTTPA**s **1**, **3**, **4**, and **6**, water–vapor adsorption measurements were performed at 298 K. All **pTTPA**s exhibited a steep uptake of water vapor at RHs of ≥80%. As shown in the previous study,^[^ ^9^ ^]^ in highly hydrophobic pores, because of the weak adsorbate–adsorbent interaction and the difficulty of forming clusters which is the first step in water adsorption, the adsorption process tends to begin at high relative humidity. In the same way, the **pTTPA**s obtained in this study do not have highly polar functional groups in their skeletons, in other words, hydrophobic pore surfaces, and therefore water adsorption begins at high relative humidity. The amount of water vapor adsorbed increased with increasing ***S*_BET_**, reaching a maximum of 52.7 mmol g^−1^ for **pTTPA 6**. This adsorption capacity corresponds to 42.6 water molecules per **TTPA** unit. In **pTTPA6**, the second stage of adsorption was observed at almost 100% RH, indicating that **pTTPA6** has larger pores than other **pTTPA**s, in addition to pores of a comparable size.^[^ ^9^ ^]^ The significantly higher water‐adsorption capacities of **pTTPA**s **3**, **4**, and **6** than those of **pTTPA 1** are attributed to the onset of capillary condensation at an RH of ≈80%. In **pTTPA**s **3**, **4**, and **6**, the higher ***S*_BET_** exposes more N atoms on the pore surface, which serve as adsorption sites for water molecules. This facilitates the formation of water clusters and subsequent pore filling through cluster aggregation.^[^ ^32^ ^]^ These results support that a higher ***S*_BET_** enhances the adsorption capacity of water, which acts as a conducting media, assisting in the conduction of proton carriers,^[^ ^33^ ^]^ and leading to the emergence of proton conductivity. As shown in Table 2, to clarify the reason for the lower proton conductivity in **pTTPA 6**, which has higher water–vapor adsorption than that of **pTTPA 4**, we calculated the micropore surface area (***S*_micro_**) and micropore volume (***V*_micro_**) for pores with diameters of ≤ 2 nm using the t‐plot method. The ***S*_micro_** values generally correlate with the ***S*_BET_** values, with **pTTPA 4** exhibiting a particularly high ***S*_micro_** compared to its ***S*_BET_**. Moreover, **pTTPA 4** exhibits the highest ***V*_micro_** among the **pTTPA**s. Therefore, the high microporosity of **pTTPA 4** could effectively lower the activation energy for self‐dissociation of water molecules by the confinement effect, leading to higher proton conductivity than **pTTPA 6**.

Previous studies have shown that proton conductivity can be improved by tuning porosity^[^ ^34^ ^]^ or controlling linker defects.^[^ ^35^ ^]^ However, these approaches have yielded only modest improvements. By contrast, we achieved a substantial enhancement of proton conductivity by four orders of magnitude solely by increasing the ***S*_BET_** while maintaining the same chemical structure. This demonstrates the effectiveness of the iodine‐based chemical polymerization method for synthesizing highly porous amorphous **POP**s that exhibit proton conductivity. Therefore, this approach can potentially transform amorphous **POP**s, conventionally considered proton insulators, into proton conductors, and maximize the proton conductivity of previously synthesized proton‐conductive amorphous **POP**s.

### 2.3 CO_2_ Adsorption of pTTPAs

**Figure 6:**
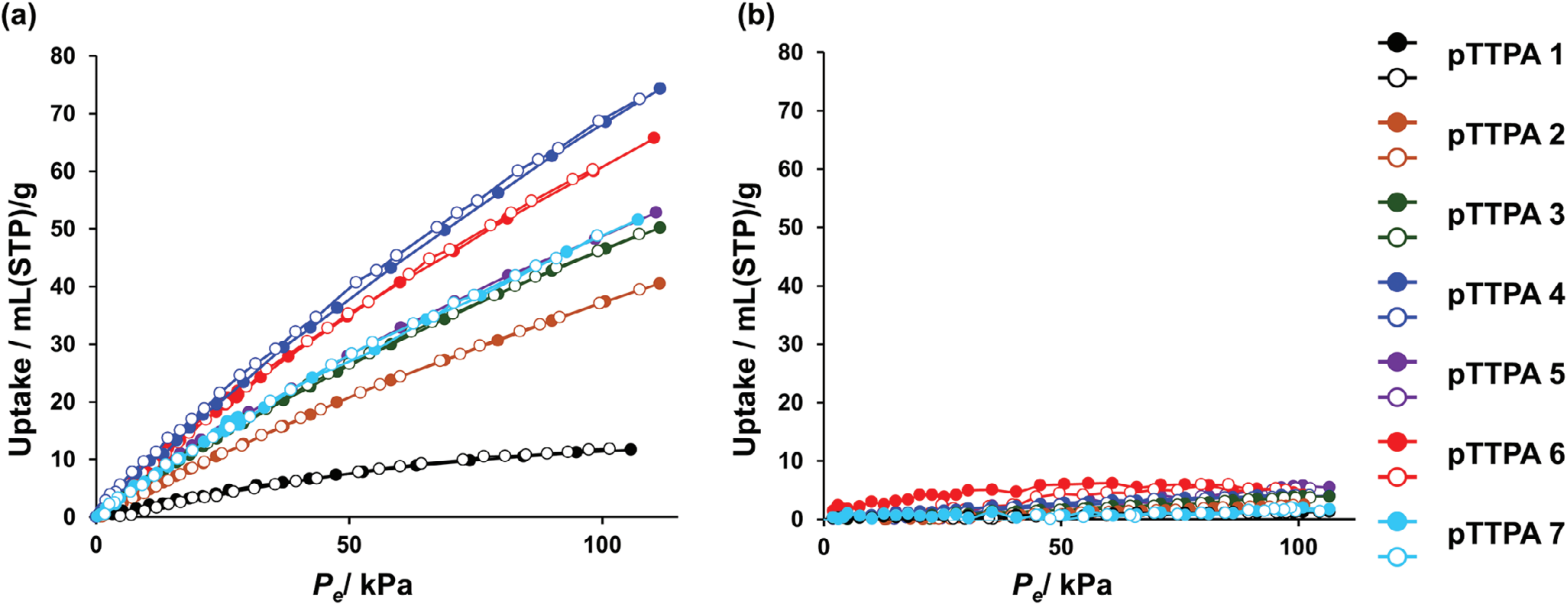
a) CO_2_ and b) N_2_ adsorption isotherms of **pTTPA 1** (black), **pTTPA 2** (brown), **pTTPA 3** (green), **pTTPA 4** (blue), **pTTPA 5** (purple), **pTTPA 6** (red), and **pTTPA 7** (light blue), measured at 298 K. Solid and hollow symbols denote the adsorption and desorption isotherms, respectively.

**pTTPA**s, which contain N atoms as one of the heteroatoms^[^ ^1b^ ^]^ and feature high porosity, are also promising adsorbents for CO_2_. As shown in **Figure** **6**, CO_2_ and N_2_ adsorption measurements for **pTTPA**s **1–7** were conducted at 298 K; the adsorption amounts at 110 kPa are summarized in **Table** **3**. Although the ***S*_BET_** of **pTTPA 4** was not the largest among the **pTTPA**s, **pTTPA 4** exhibited the highest CO_2_ adsorption of 3.31 mmol g^−1^. As shown in Table 3, the selectivity of CO_2_ adsorption over N_2_ was also the highest for **pTTPA 4**, at 18.6. Furthermore, as shown in Table S4 (Supporting Information), this CO_2_ adsorption amount is the highest among the other triphenylamine‐based amorphous **POP**s containing no basic amines that could serve as chemisorption sites for CO_2_. The amount of CO_2_ adsorption at low pressures, such as below atmospheric pressure, increases as the surface area and volume of the micropore increase.^[^ ^36^ ^]^ Therefore, as shown in Table 2, **pTTPA 4**, which has relatively high microporosity, exhibited the highest CO_2_ adsorption capacity among the **pTTPA**s. In this study, **pTTPA 4**, which had a smaller micropore surface area (***S*_micro_**) than that of **pTTPA 6**, exhibited the highest CO_2_ adsorption amount and CO_2_ selectivity, and therefore the micropore volume (***V*_micro_**) mainly governed the CO_2_ adsorption amount and CO_2_ selectivity. Furthermore, the abundance of micropores favors the adsorption of CO_2_ over N_2_ owing to the molecular sieving effect,^[^ ^37^ ^]^ as CO_2_ has a smaller dynamic molecular diameter (3.30Å^[^ ^38^ ^]^) than N_2_ (3.64Å^[^ ^38^ ^]^). This also explains the high CO_2_ selectivity of **pTTPA 4**. These results show that improving microporosity increases CO_2_ adsorption capacity.

**Table 3:** CO_2_ and N_2_ adsorption amount of **pTTPA**s **1–7**.

Sample	*V*_CO2_ ^a)^ [mL g^−1^]	*V*_N2_ ^b^ ^)^ [mL g^−1^]	*V*_CO2_/*V*_N2_
pTTPA 1	11.7	1.30	1.67
pTTPA 2	40.5	2.47	16.4
pTTPA 3	50.2	3.90	12.9
pTTPA 4	74.3	3.99	18.6
pTTPA 5	52.8	5.45	9.69
pTTPA 6	65.7	4.13	15.9
pTTPA 7	51.6	2.80	18.4

^a)^ CO_2_ adsorption amount at 110 kPa;

^b)^ N_2_ adsorption amount at 110 kPa.

## 3 Conclusion

In this study, an iodine‐based chemical polymerization method was employed to synthesize **pTTPA** without impurities by polymerizing a monomer comprising three triphenylamine units connected by a central benzene ring. The resulting **pTTPA** exhibited the largest **BET** specific surface area among reported amorphous **POP**s containing triphenylamine. Despite possessing the same chemical structure, the water–vapor adsorption capacity of **pTTPA** increased significantly with increasing porosity, leading to the emergence of proton conductivity. This result demonstrates that this approach can potentially transform amorphous **POP**s, conventionally considered proton insulators, into proton conductors, and maximize the proton conductivity of previously synthesized proton‐conductive amorphous **POP**s. Furthermore, **pTTPA** exhibited a high CO₂ adsorption capacity. These findings demonstrate that maximizing the porosity of amorphous **POP**s without impurities through iodine‐based chemical polymerization promotes the development of novel functionalities in these materials.

## Conflict of Interest

The authors declare no conflict of interest.

## Supporting information



Supporting Information

## Data Availability

The data that support the findings of this study are available in the supplementary material of this article.
