# Supplementary material for: Triphenylamine‐Based Porous Organic Polymers with High Porosity: their High Carbon‐Dioxide Adsorption and Proton‐Conductivity Emergence
Source: Small. 2025 Feb 17;21(31):2410794. doi: 10.1002/smll.202410794 (PMC12332806; doi:10.1002/smll.202410794)
Supplement: Supplementary file 1 — Supporting Information [file SMLL-21-2410794-s001.docx]

**Triphenylamine-Based Porous Organic Polymers with High Porosity: Their High Carbon Dioxide Adsorption and Proton Conductivity Emergence**

Kohei Okubo,^†[a]^ Showa Kitajima,^†[a]^ Hitoshi Kasai,^[a]^ and Kouki Oka*^[a, b, c]^

[a] Institute of Multidisciplinary Research for Advanced Materials,

Tohoku University

2-1-1 Katahira, Aoba-ku, Sendai, Miyagi 980-8577, Japan

E-mail: oka@tohoku.ac.jp (Kouki Oka)

[b] Carbon Recycling Energy Research Center,

Ibaraki University

4-12-1 Nakanarusawa, Hitachi, Ibaraki 316-8511, Japan

[c] Deuterium Science Research Unit, Center for the Promotion of Interdisciplinary Education and Research,

Kyoto University

Yoshida, Sakyo-ku, Kyoto 606-8501, Japan

^†^Kohei Okubo and Showa Kitajima contributed equally to this work.

1. **Measurements**

Infrared (**IR**) spectra were recorded on a Shimadzu IRSpirit equipped with an attenuated total reflectance (**ATR**) accessory. Raman spectra were recorded on a Renishaw inVia confocal Raman microscope system with a 532 nm laser. Matrix-assisted laser desorption ionization-time of flight mass spectrometry (**MALDI-TOF MS**) was performed on a Shimadzu MALDI-8030 instrument. Thermogravimetric analyses (**TG**) were performed with a Shimadzu DTG-60A at a heating rate of 1 °C/min under a N_2_ atmosphere. Scanning electron microscopy with energy-dispersive X-ray (**SEM-EDX**) spectra (accelerating voltage of 15 kV) were obtained using a HITACHI SU6600. X-ray diffraction (**XRD**) analyses were performed with a Rigaku SmartLab X-ray diffraction system. UV-vis spectra were recorded on a Shimadzu UV-1900i spectrophotometer. Gas adsorption measurements were performed on a MicrotracBEL BELSORP-max X instrument. Nitrogen adsorption isotherms were collected at 77 and 298 K. Carbon dioxide adsorption isotherms were collected at 298 K. Before all measurements, samples were dried under reduced pressure at 353 K for 3 h.

1. **Materials.**

1,3,5-Tris[4-(diphenylamino)phenyl]benzene (**TTPA**) and iodine were purchased from Tokyo Chemical Industry. 1,2-Dichloroethane and ethanol were purchased from Nacalai Tesque. All chemicals were used as received without further purification.

1. **pTTPA 1 Synthesis**

**TTPA** (249.5 mg, 0.309 mmol) and iodine (772.6 mg, 3.04 mmol) were added to 1,2-dichloroethane (10 mL) and the mixture was stirred for 20 h at 80 °C. The reaction mixture was then poured into ethanol. The resulting precipitate was collected via filtration and washed with ethanol, which yielded a pale-yellow powder (237.8 mg).

1. **pTTPA 2** **synthesis**

**TTPA** (255.4 mg, 0.316 mmol) and iodine (3157.5 mg, 12.4 mmol) were added to 1,2-dichloroethane (10 mL) and the mixture was stirred for 20 h at 80 °C. The reaction mixture was then poured into ethanol. The resulting precipitate was collected via filtration and washed with ethanol, which yielded a pale-yellow powder (241.6 mg).

1. **pTTPA 3** **synthesis**

**TTPA** (248.0 mg, 0.307 mmol) and iodine (7888.6 mg, 31.1 mmol) were added to 1,2-dichloroethane (10 mL), and the mixture was stirred for 20 h at 80 °C. The reaction mixture was then poured into ethanol. The resulting precipitate was collected via filtration and washed with ethanol, which yielded a light-brown powder (237.0 mg).

1. **pTTPA 4 synthesis**

**TTPA** (236.9 mg, 0.293 mmol) and iodine (15660 mg, 61.7 mmol) were added to 1,2-dichloroethane (10 mL), and the mixture was stirred for 20 h at 80 °C. The reaction mixture was then poured into ethanol. The resulting precipitate was collected via filtration and washed with ethanol, which yielded a light-brown powder (237.0 mg).

1. **pTTPA 5 synthesis**

**TTPA** (50.03 mg, 0.0619 mmol) and iodine (7823.6 mg, 30.8 mmol) were added to 1,2-dichloroethane (2 mL), and the mixture was stirred for 20 h at 80 °C. The reaction mixture was then poured into ethanol. The resulting precipitate was collected via filtration and washed with ethanol, which yielded a light-brown powder (39.60 mg).

1. **pTTPA 6 synthesis**

**TTPA** (49.3 mg, 0.0610 mmol) and iodine (3123.0 mg, 12.3 mmol) were added to 1,2-dichloroethane (2 mL), and the mixture was stirred for 20 h at 90 °C. The reaction mixture was then poured into ethanol. The resulting precipitate was collected via filtration and washed with ethanol, which yielded a light-brown powder (42.43 mg).

1. **pTTPA 7** **synthesis**

**TTPA** (52.81 mg, 0.0654 mmol) and iodine (7925.1 mg, 31.2 mmol) were added to 1,2-dichloroethane (2 mL), and the mixture was stirred for 20 h at 90 °C. The reaction mixture was then poured into ethanol. The resulting precipitate was collected via filtration and washed with ethanol, which yielded a light-brown powder (41.23 mg).


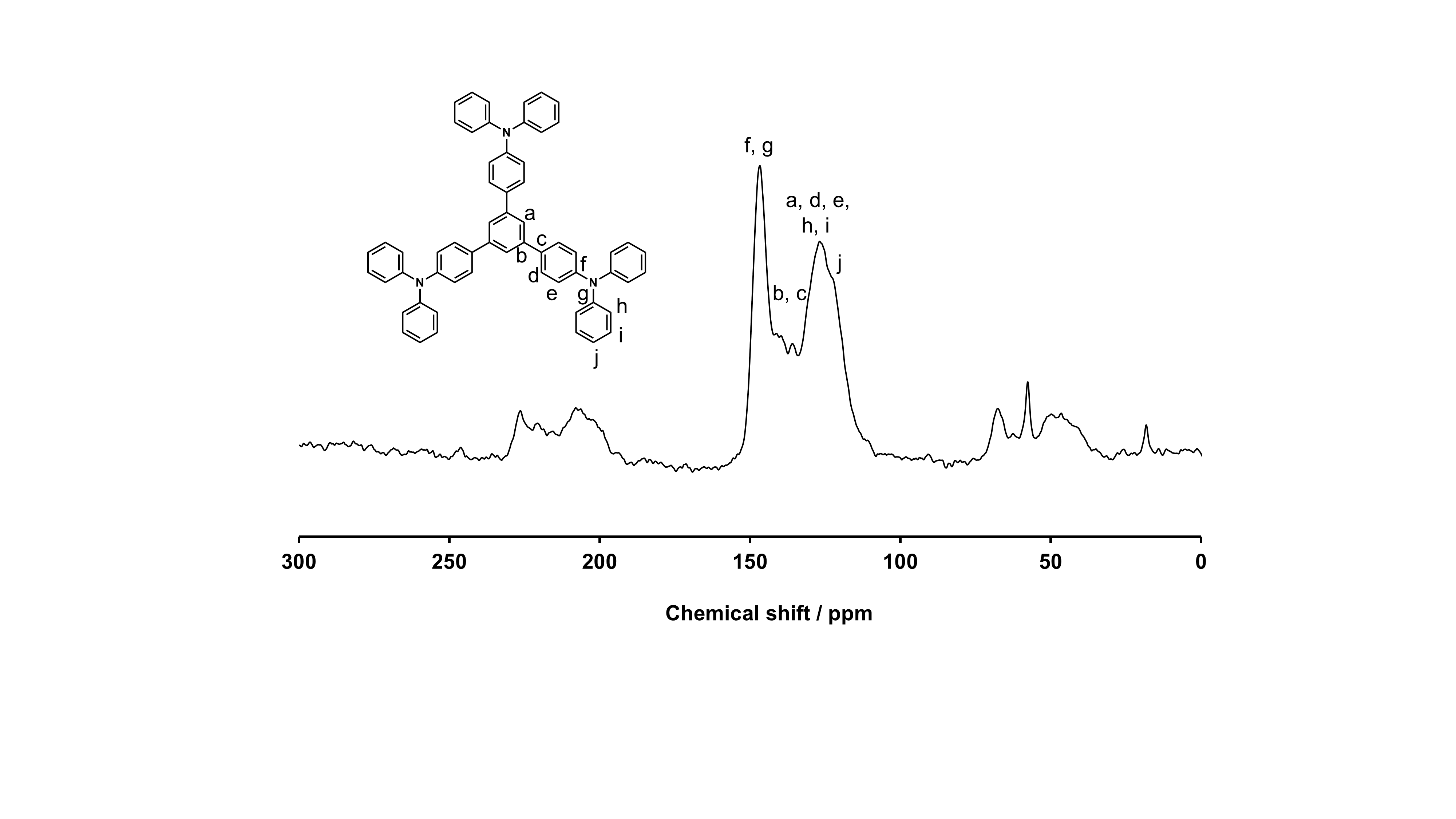


**Figure S1.** Solid-state ^13^C **NMR** spectrum of **TTPA**. Broad peaks around 50 and 210 ppm indicate spinning sidebands. The sharp peak around 10 ppm indicates impurities originally contained in the reagent, which were removed during the purification process after polymerization.


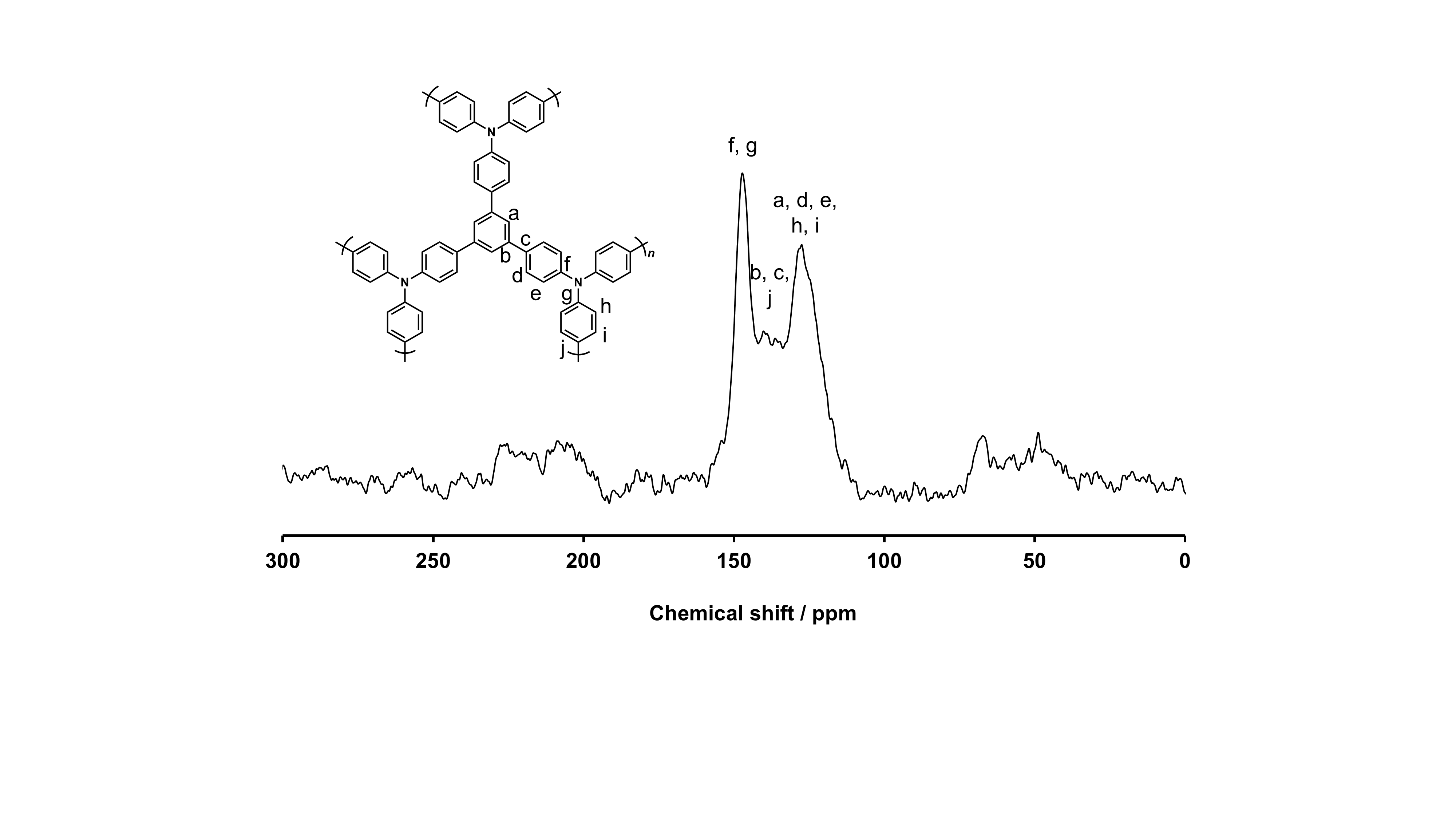


**Figure S2.** Solid-state ^13^C **NMR** spectrum of **pTTPA 1**. Broad peaks around 50 and 210 ppm indicate spinning sidebands.


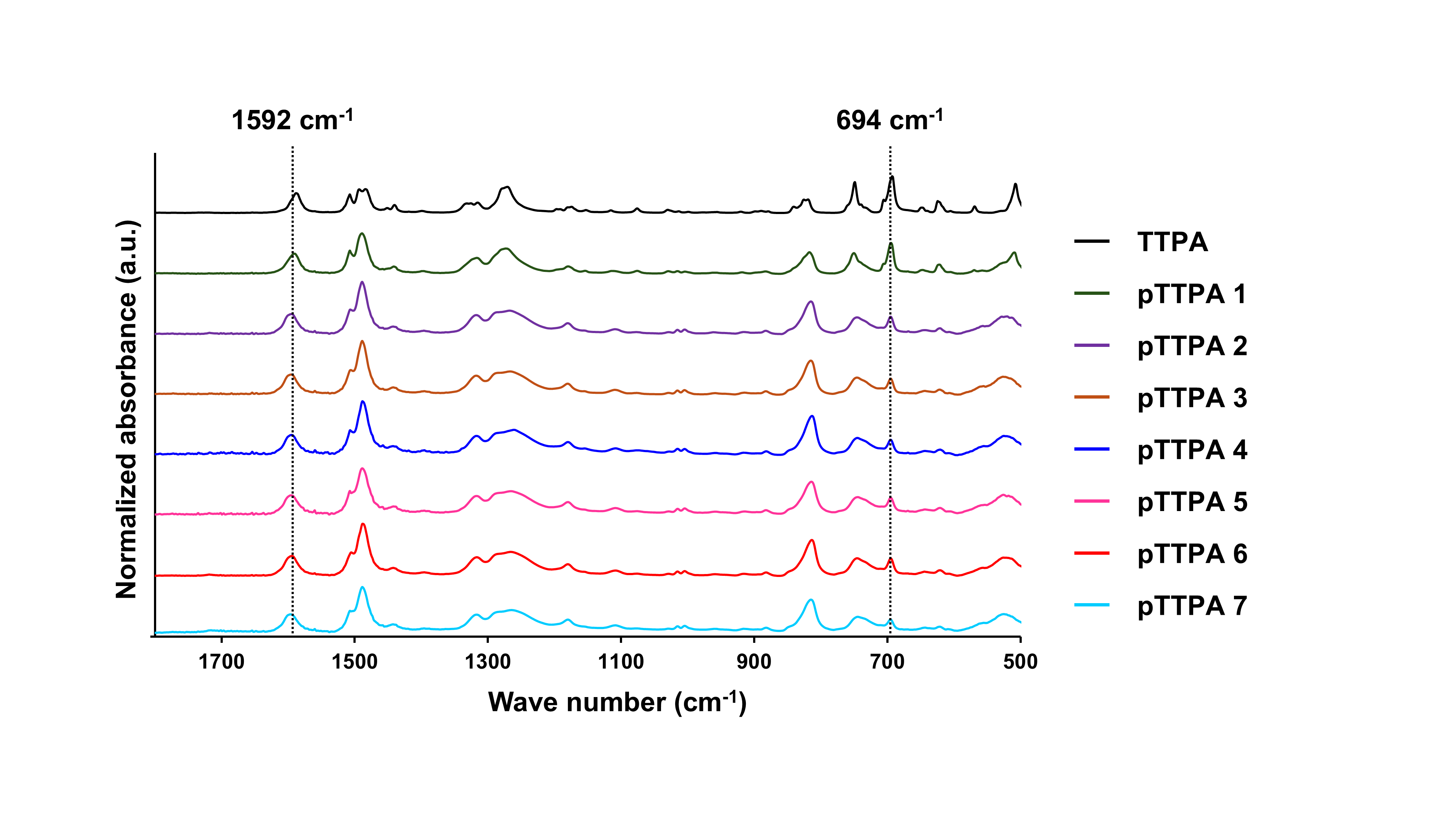


**Figure S3. IR** absorbance spectra of **TTPA** and **pTTPA**s **1–7**, normalized by the peaks around 1592 cm^-1^. The peaks at 694 and 1590 cm^-1^ correspond to the C–H bending vibration of monosubstituted benzene and C=C stretching vibration of benzene, respectively.


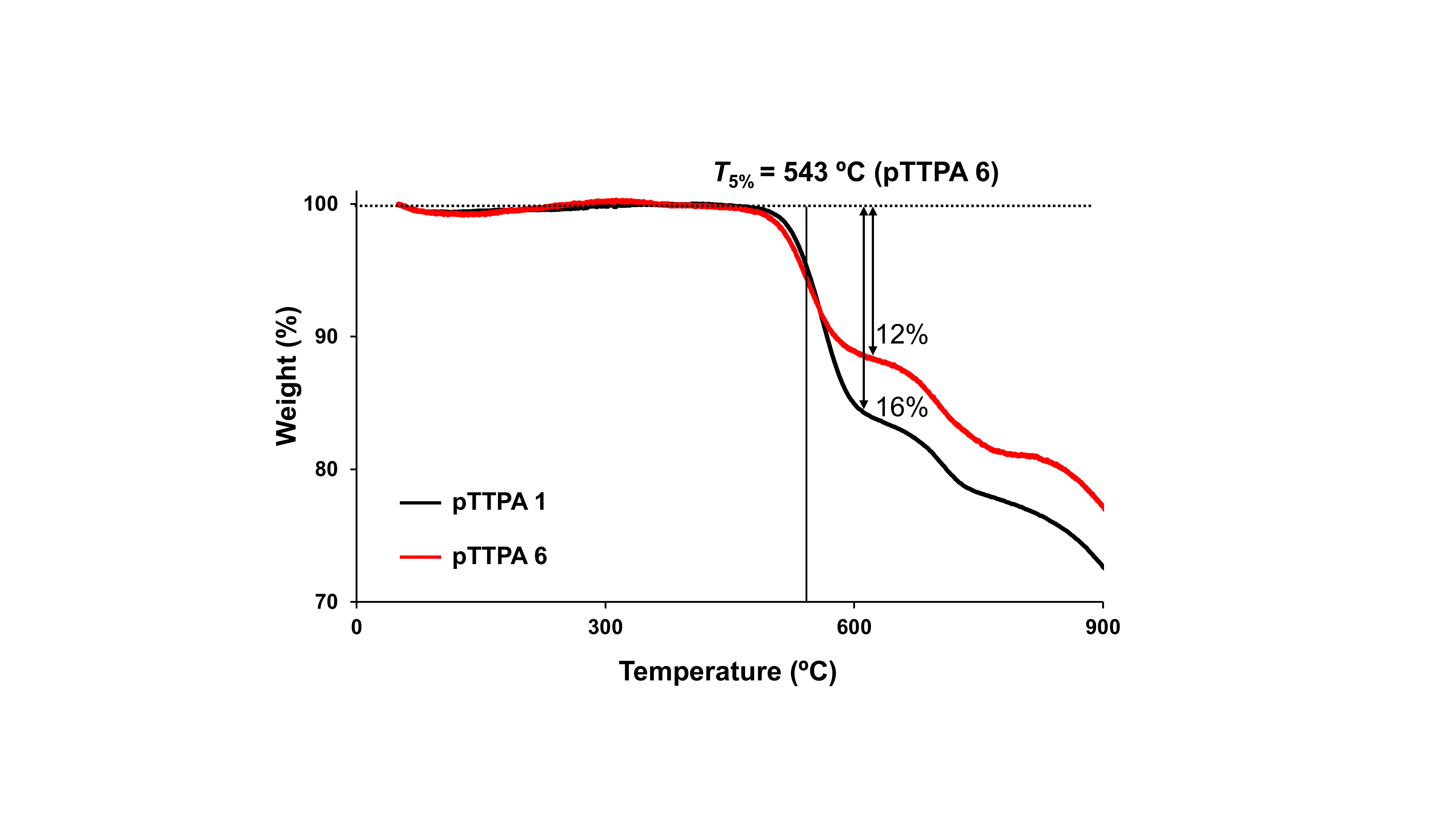


**Figure S4. TG** curves of **pTTPA 1** (black) and **pTTPA 6** (red).


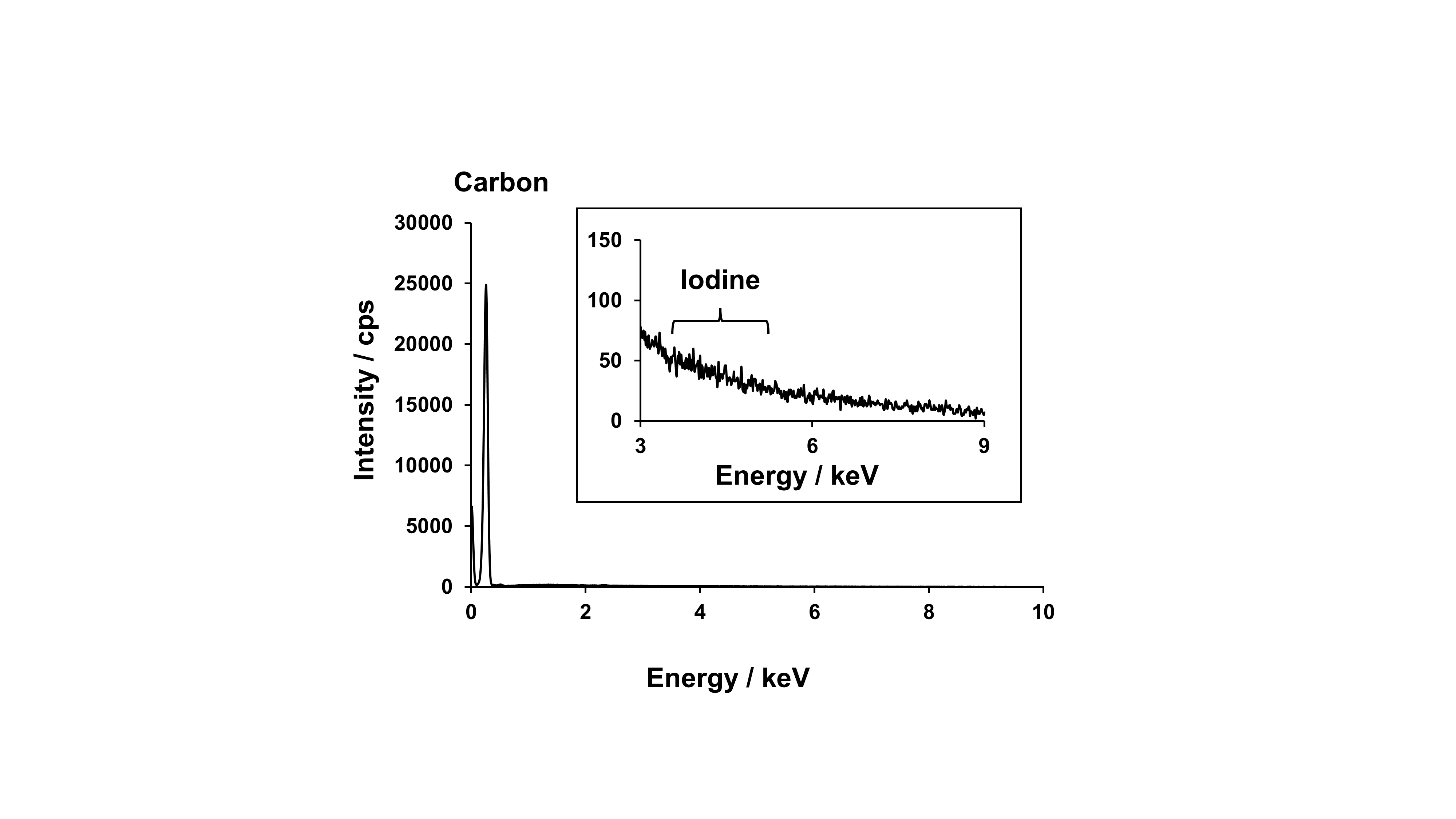


**Figure S5. SEM-EDX** spectrum of **pTTPA 1**.


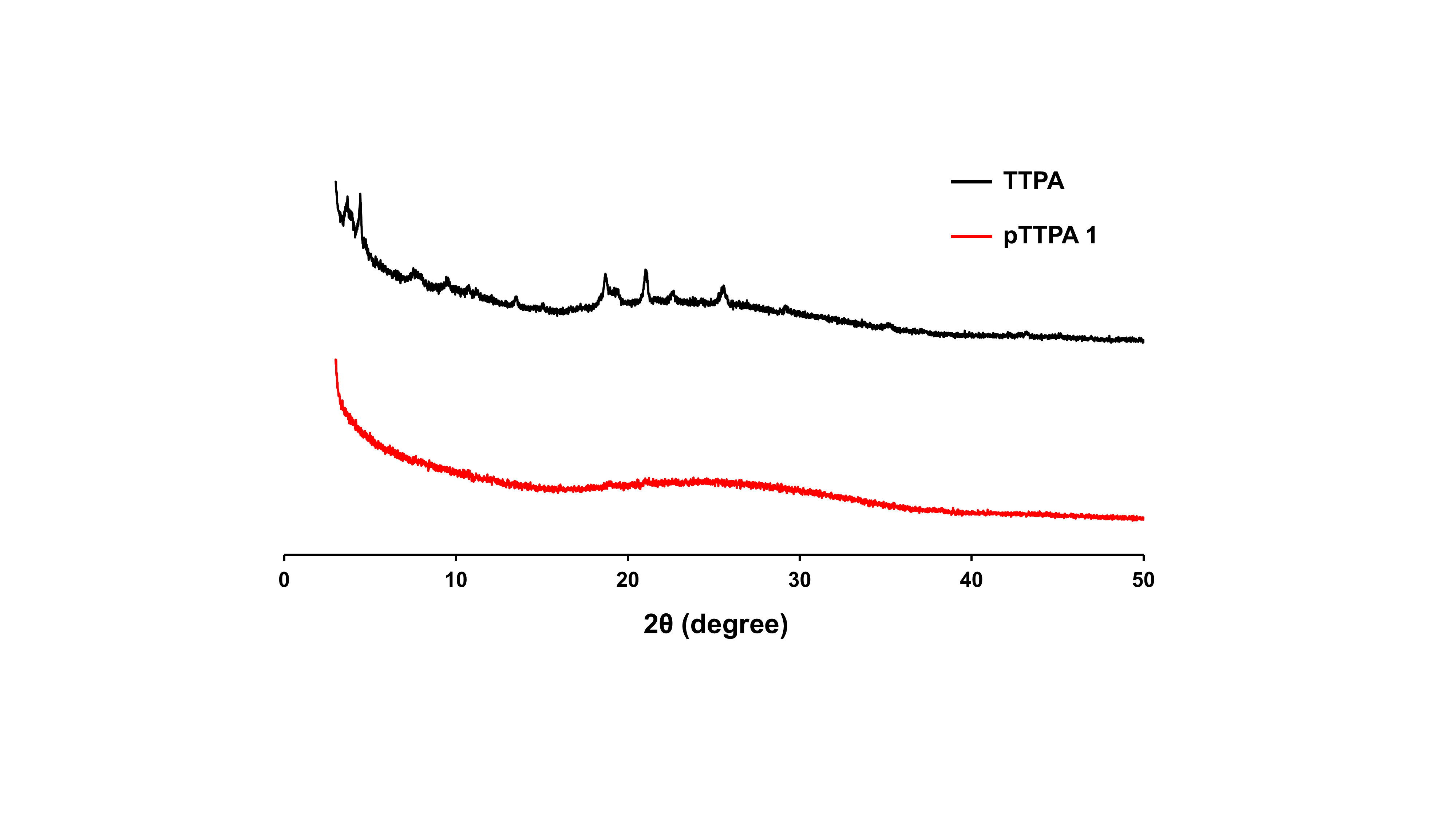


**Figure S6. XRD** patterns of **TTPA** (black) and **pTTPA 1** (red).


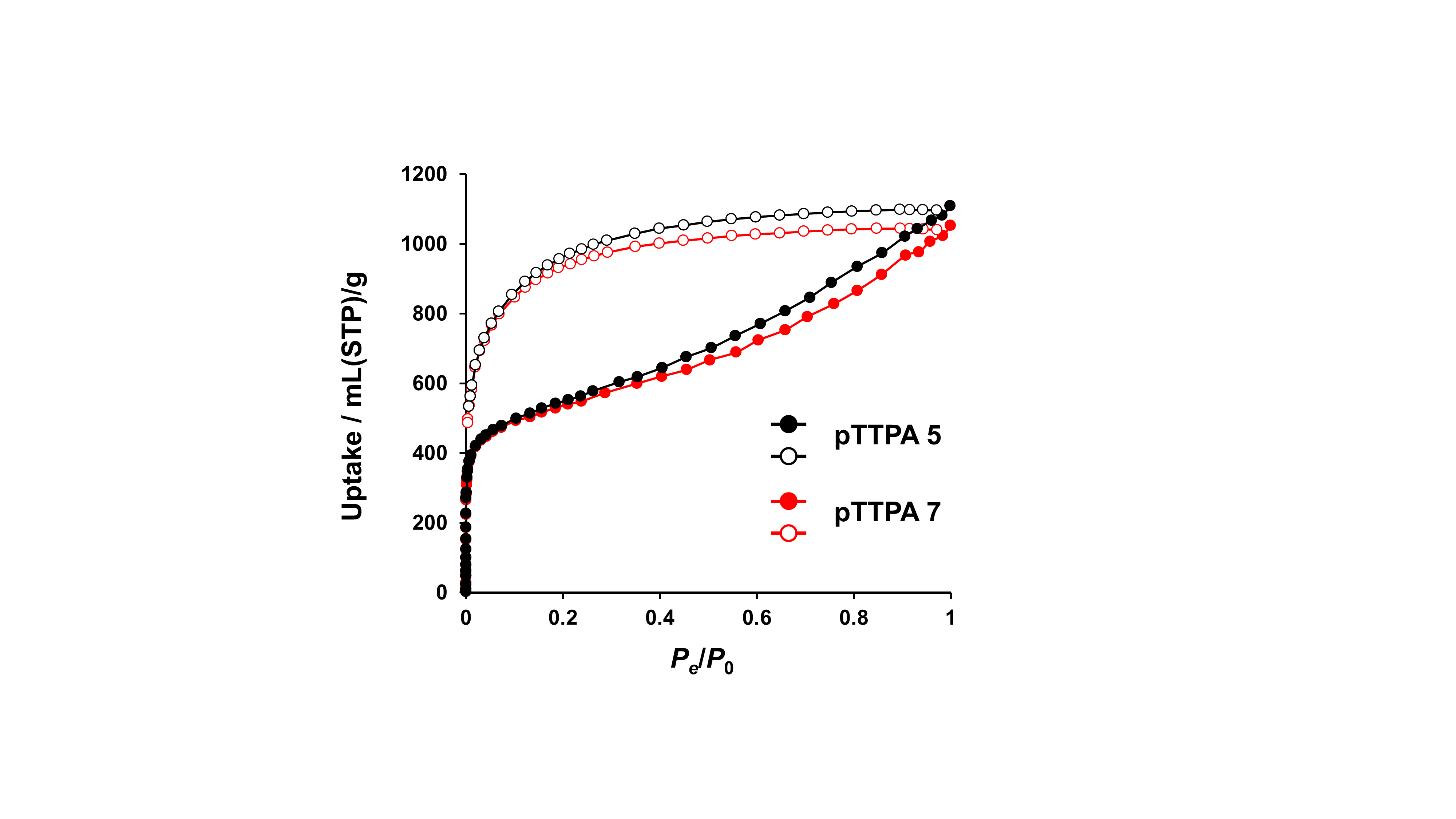


**Figure S7.** N_2_ adsorption isotherms of **pTTPA 5** (black) and **pTTPA 7** (red) measured at 77 K. Solid and hollow symbols denote the adsorption and desorption isotherms, respectively.


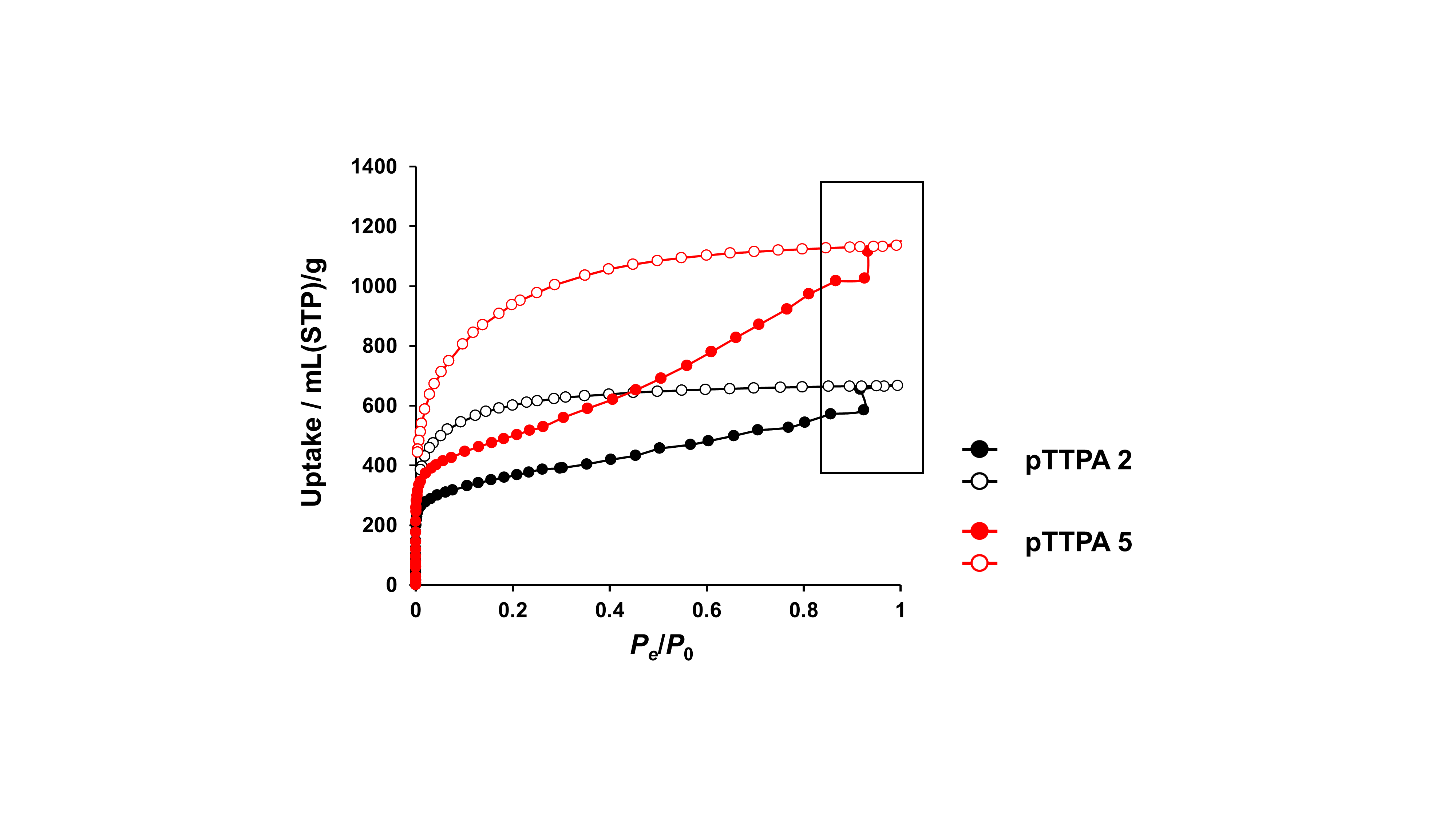


**Figure S8.** N_2_ adsorption isotherms of **pTTPA 2** (black) and **pTTPA 5** (red) measured at 77 K. Solid and hollow symbols denote the adsorption and desorption isotherms, respectively.


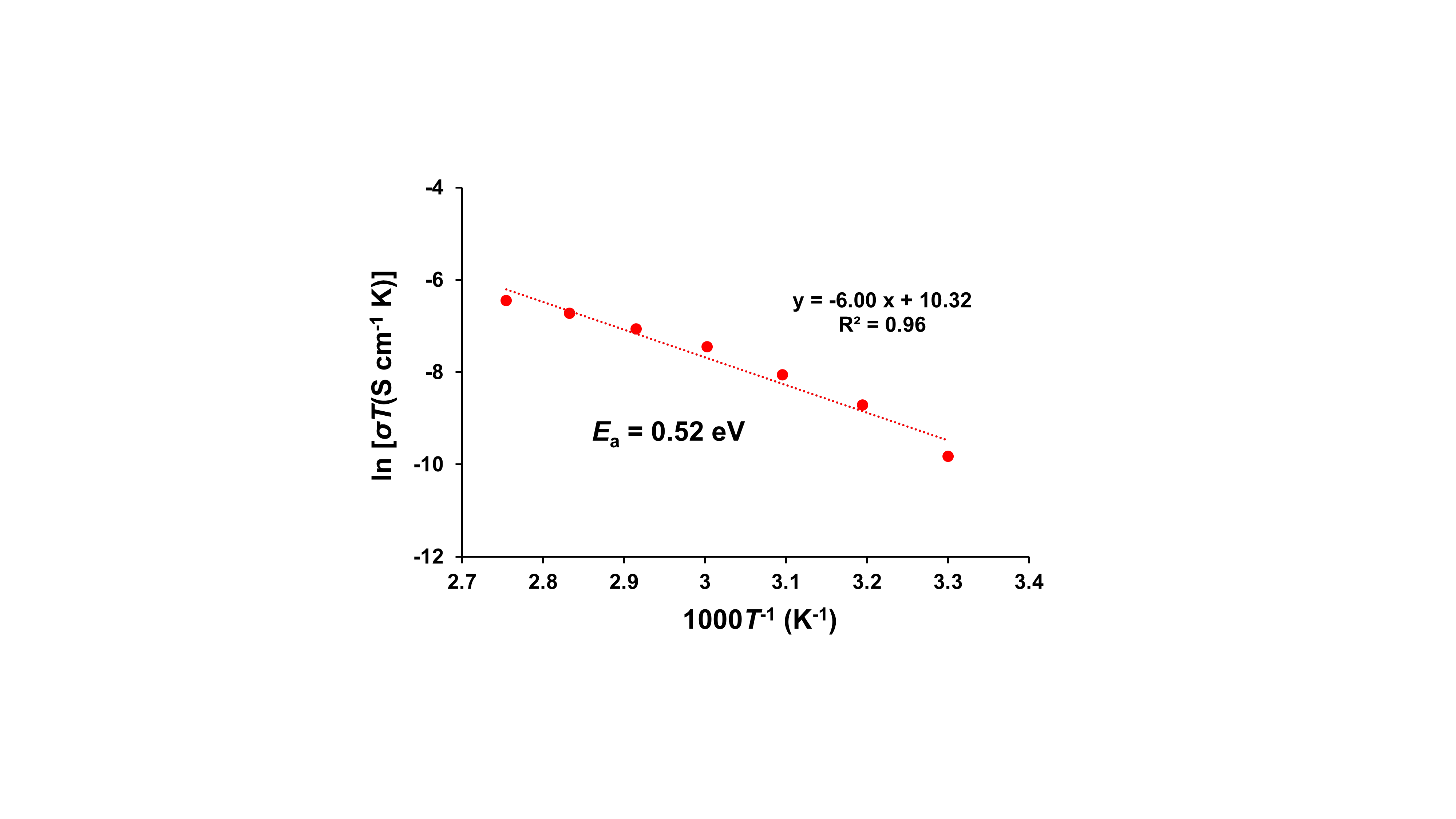


**Figure S9.** Arrhenius plots for proton conductivity of **pTTPA 4** at 30–90 °C under 95% RH.

**Table S1.** Raman spectra assignments of **pTTPA**.

| Assignments | Raman shift (cm^-1^) |
| --- | --- |
| Ring stretching | 777 |
| Ring deformation | 818 |
| C-H bending | 996 |
| Ring deformation | 1029 |
| C-N stretching | 1169 |
| C-C stretching | 1289 |
| C-H in-plane bending | 1349 |
| C＝C stretching | 1610 |

**Table S2.** Absorbance of the 694 cm^-1^ peak corresponding to the C–H bending vibration of monosubstituted benzene from the **IR** spectra of **TTPA** and **pTTPA**s **1–7**, percentage of remaining C-H bonds of monosubstituted benzene, and polymerization degree.

| Sample | Absorbance of the peak at 694 cm^-1^  / a.u. | Percentage of remaining C-H bonds of monosubstituted benzene / % | Degree of polymerization/ % |
| --- | --- | --- | --- |
| **TTPA** | 1.840 | 100 | 0 |
| **pTTPA 1** | 1.531 | 83 | 17 |
| **pTTPA 2** | 0.864 | 47 | 53 |
| **pTTPA 3** | 0.793 | 43 | 57 |
| **pTTPA 4** | 0.757 | 41 | 59 |
| **pTTPA 5** | 0.737 | 40 | 60 |
| **pTTPA 6** | 0.736 | 40 | 60 |
| **pTTPA 7** | 0.736 | 40 | 60 |

**Table S3. BET** surface area of **pTTPA**s **1–7** in this work and previously reported triphenylamine-based amorphous **POP**s.

| Name | ***S*_BET_**  / m^2^g^-1^ | Reference |
| --- | --- | --- |
| **pTTPA 1** | **315.93** | **This work** |
| **pTTPA 2** | **1318.2** | **This work** |
| **pTTPA 3** | **1579.8** | **This work** |
| **pTTPA 4** | **1802.8** | **This work** |
| **pTTPA 5** | **1976.6** | **This work** |
| **pTTPA 6** | **2134.6** | **This work** |
| **pTTPA 7** | **1984.1** | **This work** |
| HCP1 | 534.5 | [1] |
| HCP3 | 199.9 | [1] |
| HCP4 | 10.8 | [1] |
| HCP6 | 30.3 | [1] |
| TPPA-CC | 742 | [2] |
| TPAA-DMB | 883 | [2] |
| TPPA-DMM | 830 | [2] |
| MAPOP-4 | 820 | [3] |
| FCDTPA | 871 | [4] |
| TBAL-POP-1 | 712 | [5] |
| TBAL-POP-2 | 1104 | [5] |
| TMP-1 | 452 | [6] |
| TMP-2 | 613 | [6] |
| TMP-3 | 709 | [6] |
| TE-POP | 75 | [7] |
| ANOP-6 | 657 | [8] |
| ANOP-7 | 697 | [8] |
| DPT-HPP | 1230 | [9] |
| TPA–DHTP–BZ POP | 195 | [10] |
| poly (TPA–DHTP–BZ) POP | 40 | [10] |
| TPOA-F | 1179 | [11] |
| TPOA-OH | 565 | [11] |
| TPA-CH POP | 822 | [12] |
| TPA-CH POP-SH | 850 | [12] |
| CNOP-1 | 1374 | [13] |
| PTPA-1 | 841 | [14] |
| PTPA-2 | 1216 | [14] |
| PTPA-3 | 1201 | [14] |
| PTPA-4 | 1543 | [14] |
| PTPA-5 | 1365 | [14] |
| PHCTF-8(400) | 1188 | [15] |
| PHCTF-8(500) | 1197 | [15] |
| PHCTF-8(550) | 1340 | [15] |
| PHCTF-8(600) | 1400 | [15] |
| PHCTF-8(650) | 1471 | [15] |
| PHCTF-8(700) | 1665 | [15] |
| TPA-PTCDA-MA | 555 | [16] |
| TPA-PMDA-MA | 557 | [16] |
| aTMCOP | 1104 | [17] |
| TPA-TMC-MA | 448 | [18] |
| TPA-TC-MA | 454 | [18] |
| ANOP-M | 1272 | [19] |
| ANOP-A | 1052 | [19] |
| YPTPA | 1557 | [20] |
| SPTPA | 544 | [20] |
| PPTPA | 1437 | [21] |
| JUC-Z2 | 2081 | [22] |
| PAN-B | 1254 | [23] |
| PAN-T | 1273 | [23] |
| TPAPOPs 1 | 50 | [24] |
| TPAPOPs 2 | 20 | [24] |
| TPAPOPs 3 | 13 | [24] |
| PBPn25 | 1250 | [25] |
| PTATE2 | 20.3 | [26] |
| PTATE4 | 13.6 | [26] |
| IPN-CSU-1 | 584.3 | [27] |
| IPN-CSU-2 | 708.3 | [27] |
| POP-TPA | 259.2 | [28] |
| POP-I | 563 | [29] |
| POP-II | 792 | [29] |
| PPN1 | 1392 | [30] |
| PPN2 | 1081 | [30] |
| PAF-159 | 814 | [31] |
| PBP-N-25 | 1362 | [32] |
| PBP-N-50 | 1338 | [32] |
| POPCo1 | 657 | [33] |
| POPCo2 | 669 | [33] |
| POPCo3 | 688 | [33] |
| PPN | 1165 | [34] |
| PPTPA-1 | 1437 | [21] |
| TB-POP | 252 | [35] |
| TPT | 532.8 | [36] |
| Co_3_O_4_@POP | 582 | [37] |
| CoS_x_@POP | 489 | [37] |
| Ni@T-POP | 190 | [38] |
| CE-Py POP | 28 | [39] |
| TPA-Py POP | 70 | [40] |
| TPA-POP | 354 | [41] |
| TATPA-POP | 400.8 | [42] |
| TPA-Th-POP | 73 | [43] |
| TPA-DPA PPK | 675 | [44] |
| PAN-L | 507 | [45] |
| PAN-L-Br | 322 | [45] |

[a] CO_2_ adsorption amount under 110 kPa at 298 K.

**Table S4.** CO_2_ adsorption amount of **pTTPA**s **1–7** in this work and previously reported triphenylamine-based amorphous **POP**s containing no basic amines.

| Name | ***V*_CO2_** ^[a]^  / mL g^-1^ | Reference |
| --- | --- | --- |
| **pTTPA 1** | **11.7** | **This work** |
| **pTTPA 2** | **40.5** | **This work** |
| **pTTPA 3** | **50.2** | **This work** |
| **pTTPA 4** | **74.3** | **This work** |
| **pTTPA 5** | **52.8** | **This work** |
| **pTTPA 6** | **65.7** | **This work** |
| **pTTPA 7** | **51.6** | **This work** |
| HCP1 | 35.1 | [1] |
| HCP3 | 32.7 | [1] |
| HCP4 | 16.2 | [1] |
| HCP6 | 9.5 | [1] |
| TPAA-DMB | 39.6 | [2] |
| TPPA-DMM | 37.6 | [2] |
| MAPOP-4 | 40.2 | [3] |
| FCDTPA | 33.8 | [4] |
| TPA–DHTP–BZ POP | 21.7 | [10] |
| TPA-CH POP | 12.5 | [12] |
| TPA-CH POP-SH | 32.7 | [12] |
| CNOP-1 | 48.8 | [13] |
| PTPA-1 | 32.9 | [14] |
| PTPA-2 | 29.8 | [14] |
| PTPA-3 | 28.9 | [14] |
| PTPA-4 | 46.8 | [14] |
| PTPA-5 | 26.9 | [14] |
| TPA-PTCDA-MA | 50.0 | [16] |
| TPA-PMDA-MA | 49.0 | [16] |
| ANOP-M | 40.6 | [19] |
| ANOP-A | 39.3 | [19] |
| YPTPA | 34.9 | [20] |
| SPTPA | 18.4 | [20] |

[a] CO_2_ adsorption amount under 110 kPa at 298 K.

[1] L. Shao, Y. Sang, N. Liu, Q. Wei, F. Wang, P. Zhan, W. Luo, J. Huang, J. Chen, *Separation and Purification Technology* **2021**, *262*.

[2] E. Cucu, E. Dalkılıç, R. Altundas, A. E. Sadak, *Microporous and Mesoporous Materials* **2022**, *330*.

[3] X. Ding, H. Li, Y.-C. Zhao, B.-H. Han, *Polymer Chemistry* **2015**, *6*, 5305-5312.

[4] X. Yang, M. Yu, Y. Zhao, C. Zhang, X. Wang, J.-X. Jiang, *J. Mater. Chem. A* **2014**, *2*, 15139-15145.

[5] D. Chakraborty, R. Chatterjee, S. Mondal, S. K. Das, V. Amoli, M. Cho, A. Bhaumik, *ACS Appl Mater Interfaces* **2023**, *15*, 48326-48335.

[6] M. Rong, L. Yang, L. Wang, J. Yu, H. Qu, H. Liu, *J Colloid Interface Sci* **2019**, *548*, 265-274.

[7] T. Li, M. Chen, Q. Shi, J. Xiong, Y. Feng, B. Zhang, *New Journal of Chemistry* **2023**, *47*, 4239-4244.

[8] S. Tong, J. Zhu, Z. Wang, J. Yan, *ACS Applied Materials & Interfaces* **2024**, *16*, 42717-42725.

[9] M. G. Mohamed, A. F. M. El-Mahdy, T. S. Meng, M. M. Samy, S. W. Kuo, *Polymers (Basel)* **2020**, *12*.

[10] M. Ejaz, M. G. Mohamed, S.-W. Kuo, *Polymer Chemistry* **2023**, *14*, 2494-2509.

[11] Q. Liu, L. Jiang, J. Liu, L. Wei, J. Zhang, K. Yuan, *New Journal of Chemistry* **2023**, *47*, 17567-17577.

[12] M. G. Kotp, S.-W. Kuo, *Polymers* **2024**, *16*.

[13] J. Yan, Y. Tan, L. Wei, Z. Liu, Q. Wang, H. Sun, Z. Wang, D. Li, Y. Qian, S. Guo, *Industrial & Engineering Chemistry Research* **2022**, *61*, 13453-13460.

[14] D. Zhang, L. Tao, Q. Wang, T. Wang, *Polymer* **2016**, *82*, 114-120.

[15] K. Yuan, C. Liu, C. Liu, S. Zhang, G. Yu, L. Yang, F. Yang, X. Jian, *Polymer* **2018**, *151*, 65-74.

[16] L. Wang, G. Chen, Q. Xiao, D. Zhang, Y. Sang, J. Huang, *Industrial & Engineering Chemistry Research* **2020**, *59*, 19117-19125.

[17] S. K. Das, X. Wang, Z. Lai, *Microporous and Mesoporous Materials* **2018**, *255*, 76-83.

[18] L. Wang, Q. Xiao, D. Zhang, W. Kuang, J. Huang, Y. N. Liu, *ACS Appl Mater Interfaces* **2020**, *12*, 36652-36659.

[19] S. Xie, Q. Wang, J. Yan, H. Sun, D. Li, Y. Qian, Z. Wang, F. Han, S. Guo, *ACS Applied Polymer Materials* **2022**, *4*, 5449-5456.

[20] X. Yang, S. Yao, M. Yu, J. X. Jiang, *Macromol Rapid Commun* **2014**, *35*, 834-839.

[21] S. Mondal, J. Mondal, A. Bhaumik, *ChemCatChem* **2015**, *7*, 3570-3578.

[22] T. Ben, K. Shi, Y. Cui, C. Pei, Y. Zuo, H. Guo, D. Zhang, J. Xu, F. Deng, Z. Tian, S. Qiu, *Journal of Materials Chemistry* **2011**, *21*.

[23] C. Liu, M. Xia, M. Zhang, K. Yuan, F. Hu, G. Yu, X. Jian, *Polymer* **2020**, *194*.

[24] C. Yadav, V. K. Maka, S. Payra, J. N. Moorthy, *Journal of Catalysis* **2020**, *384*, 61-71.

[25] J. Lee, C. Ahn, S. Chang, J. Lee, T.-H. Bae, *Journal of Membrane Science* **2023**, *687*.

[26] Q. Cui, X. Guo, W. Zhang, W. Dong, Q. Duan, *Dyes and Pigments* **2023**, *208*.

[27] C. Ai, J. Feng, S. Yang, S. Xiong, J. Tang, G. Yu, C. Pan, *Separation and Purification Technology* **2021**, *279*.

[28] Y. Zhang, Y. Zhang, X. Luo, Q. Gao, Y. Liu, X. Wang, S. Zhou, D. Wang, P. Gu, Z. Li, *Separation and Purification Technology* **2023**, *327*.

[29] X. Song, W. Zhu, Y. Yan, H. Gao, W. Gao, W. Zhang, M. Jia, *Microporous and Mesoporous Materials* **2017**, *242*, 9-17.

[30] C. Y. Chuah, Y. Yang, T.-H. Bae, *Microporous and Mesoporous Materials* **2018**, *272*, 232-240.

[31] Y. Jing, C. Wang, B. Zhu, S. Xiao, W. Guan, S. Liu, N. Zhang, S. Wen, G. Zhu, *Angew Chem Int Ed Engl* **2024**, *63*, e202410069.

[32] Y. Yang, Q. Zhang, S. Zhang, S. Li, *RSC Advances* **2014**, *4*.

[33] M. B. Solomon, A. Rawal, J. M. Hook, S. M. Cohen, C. P. Kubiak, K. A. Jolliffe, D. M. D'Alessandro, *RSC Adv* **2018**, *8*, 24128-24142.

[34] C. Sarkar, S. C. Shit, D. Q. Dao, J. Lee, N. H. Tran, R. Singuru, K. An, D. N. Nguyen, Q. V. Le, P. N. Amaniampong, A. Drif, F. Jerome, P. T. Huyen, T. T. N. Phan, D.-V. N. Vo, N. Thanh Binh, Q. T. Trinh, M. P. Sherburne, J. Mondal, *Green Chemistry* **2020**, *22*, 2049-2068.

[35] C. Cui, Y. Tang, M. A. Ziaee, D. Tian, R. Wang, *ChemCatChem* **2018**, *10*, 1431-1437.

[36] R. Paul, C. Sarkar, Y. Yan, Q. T. Trinh, B. S. Rao, C. W. Pao, J. F. Lee, W. Liu, J. Mondal, *ChemCatChem* **2020**, *12*, 3687-3704.

[37] S. C. Shit, S. Khilari, I. Mondal, D. Pradhan, J. Mondal, *Chemistry* **2017**, *23*, 14827-14838.

[38] P. Kalita, R. Paul, C. W. Pao, R. Chatterjee, A. Bhaumik, J. Mondal, *Chem Commun (Camb)* **2023**, *59*, 5067-5070.

[39] M. G. Mohamed, W.-C. Chang, S. V. Chaganti, S. U. Sharma, J.-T. Lee, S.-W. Kuo, *Polymer Chemistry* **2023**, *14*, 4589-4601.

[40] S. Sarkar, T. K. Dutta, B. P. Mandal, A. Patra, *Chem Commun (Camb)* **2024**, *60*, 5010-5013.

[41] N. Das, R. Paul, S. Tomar, C. Biswas, S. Chakraborty, J. Mondal, *Inorg Chem* **2024**, *63*, 6092-6102.

[42] Z. Li, Y. Fu, T. Luan, K. Cheng, X. Luo, Z. Su, H. Lu, P.-Z. Li, *ACS Applied Nano Materials* **2024**, *7*, 20897-20904.

[43] M. G. Mohamed, S. V. Chaganti, M.-S. Li, M. M. Samy, S. U. Sharma, J.-T. Lee, M. H. Elsayed, H.-H. Chou, S.-W. Kuo, *ACS Applied Energy Materials* **2022**, *5*, 6442-6452.

[44] S. Mondal, N. S. Powar, R. Paul, H. Kwon, N. Das, B. M. Wong, S. I. In, J. Mondal, *ACS Appl Mater Interfaces* **2022**, *14*, 771-783.

[45] B. Zhang, J. Yan, Y. Shang, Z. Wang, *Macromolecules* **2018**, *51*, 1769-1776.
